# Direct three-dimensional segmentation of prostate glands with nnU-Net

**DOI:** 10.1117/1.JBO.29.3.036001

**Published:** 2024-03-01

**Authors:** Rui Wang, Sarah S. L. Chow, Robert B. Serafin, Weisi Xie, Qinghua Han, Elena Baraznenok, Lydia Lan, Kevin W. Bishop, Jonathan T. C. Liu

**Affiliations:** a University of Washington, Department of Mechanical Engineering, Seattle, Washington, United States; b University of Washington, Department of Bioengineering, Seattle, Washington, United States; c University of Washington, Department of Biology, Seattle, Washington, United States; d University of Washington, Department of Laboratory Medicine and Pathology, Seattle, Washington, United States

**Keywords:** computational three-dimensional pathology, gland segmentation, prostate cancer, deep learning, biomedical image processing

## Abstract

**Significance:**

In recent years, we and others have developed non-destructive methods to obtain three-dimensional (3D) pathology datasets of clinical biopsies and surgical specimens. For prostate cancer risk stratification (prognostication), standard-of-care Gleason grading is based on examining the morphology of prostate glands in thin 2D sections. This motivates us to perform 3D segmentation of prostate glands in our 3D pathology datasets for the purposes of computational analysis of 3D glandular features that could offer improved prognostic performance.

**Aim:**

To facilitate prostate cancer risk assessment, we developed a computationally efficient and accurate deep learning model for 3D gland segmentation based on open-top light-sheet microscopy datasets of human prostate biopsies stained with a fluorescent analog of hematoxylin and eosin (H&E).

**Approach:**

For 3D gland segmentation based on our H&E-analog 3D pathology datasets, we previously developed a hybrid deep learning and computer vision-based pipeline, called image translation-assisted segmentation in 3D (ITAS3D), which required a complex two-stage procedure and tedious manual optimization of parameters. To simplify this procedure, we use the 3D gland-segmentation masks previously generated by ITAS3D as training datasets for a direct end-to-end deep learning-based segmentation model, nnU-Net. The inputs to this model are 3D pathology datasets of prostate biopsies rapidly stained with an inexpensive fluorescent analog of H&E and the outputs are 3D semantic segmentation masks of the gland epithelium, gland lumen, and surrounding stromal compartments within the tissue.

**Results:**

nnU-Net demonstrates remarkable accuracy in 3D gland segmentations even with limited training data. Moreover, compared with the previous ITAS3D pipeline, nnU-Net operation is simpler and faster, and it can maintain good accuracy even with lower-resolution inputs.

**Conclusions:**

Our trained DL-based 3D segmentation model will facilitate future studies to demonstrate the value of computational 3D pathology for guiding critical treatment decisions for patients with prostate cancer.

## 1 Introduction

Prostate cancer is the most prevalent form of cancer and is the second leading cause of cancer-related deaths among men in the United States.^1^ Every year, nearly 250,000 men are diagnosed with this disease in the United States. Morbidity and mortality rates are low, but there is a fraction of prostate cancer cases that are potentially lethal and for whom aggressive treatments are warranted. To determine whether a patient requires aggressive treatment, urologists rely heavily upon the Gleason score reported by pathologists. Gleason scoring is based solely on the visual interpretation of prostate gland morphology, as seen on a few 2D histology slides. Unfortunately, there is a high level of interobserver variability associated with Gleason grading of prostate cancer^2^^,^^3^ and the Gleason scores are only moderately correlated with outcomes, particularly for patients with intermediate-grade prostate cancer.^4^ This can lead to the undertreatment of some patients,^5^ resulting in preventable metastasis and death,^6^ and overtreatment of other patients,^7^ which can lead to financial burdens and avoidable side effects, such as incontinence and impotence.^8^

A contributing factor to the limited predictive power of Gleason grading is that with conventional slide-based histopathology, only ∼1% of each prostate biopsy is viewed in the form of thin physically sectioned tissue sections mounted on glass slides. In addition to severely undersampling the biopsy specimens, by which key structures can be missed, the interpretation of complex branching-tree glandular morphologies can be misleading and ambiguous based on 2D tissue sections. Tissue destruction is a further disadvantage of conventional histology, in which valuable tissue material is no longer available for downstream assays. Nondestructive three-dimensional (3D) pathology can enable complete imaging and analysis of biopsy specimens, providing volumetric visualization and quantification of diagnostically significant microstructures while maintaining entire tissue specimens for downstream assays.^9^ We and others have shown that 3D pathology datasets can improve the characterization of the convoluted glandular structures that pathologists presently rely on for prostate cancer risk stratification.^10^^–^^17^ For instance, a gland that seems poorly formed in two dimensions (Gleason pattern 4) might actually be a tangential section of a well-formed gland (Gleason pattern 3). As a result, the cancer’s grade determined in 2D (Gleason score 3+4=7) could be downgraded (Gleason score 3+3=6) when observed in 3D, which could lead to significantly different treatment recommendations.^13^^,^^14^ However, due to the vast amount of information contained in a 3D pathology dataset of a biopsy, which is >100-fold more than a 2D whole-slide image representation, there would be great value in computational tools for efficient and consistent prognostic analyses.

In recent years, we have developed computational methods to analyze 3D pathology datasets of prostate cancer for risk stratification (i.e., prediction of biochemical recurrence outcomes). Although weakly supervised deep-learning methods are gaining popularity and are extremely powerful,^16^ there is also value in developing traditional classifiers based on intuitive “hand-crafted” features. For example, the physical insights and 3D spatial biomarkers identified through such hand-crafted machine classifiers could be of value for hypothesis generation and for explaining why 3D information can help with diagnostic determinations. We have shown that 3D glandular features, such as volume ratios, gland tortuosity, and gland curvature, can outperform analogous 2D features for prostate cancer risk stratification.^15^ We have similarly shown that 3D nuclear features are of prognostic value.^17^ These intuitive feature-based classification approaches first require accurate segmentations of diagnostically important histological structures, such as prostate glands in our case.^18^^,^^19^ This is typically achieved in one of two ways: (i) direct deep learning (DL)-based segmentation methods^20^^–^^23^ that require manually annotated training datasets, which are especially tedious and difficult to obtain in 3D,^24^ or (ii) traditional computer vision (CV) approaches based on intensity and morphology, provided that tissue structures of interest can be stained/labeled with high specificity.^11^^,^^25^^,^^26^ Although immunolabeling can provide a high degree of specificity for conventional CV-based segmentation, it is not a practical approach for clinical 3D pathology assays due to the high cost of antibodies required to stain large tissue volumes and the slow diffusion times of antibodies in thick tissues.^27^^,^^28^

As an alternative 3D segmentation approach, Xie et al. proposed a method called image translation-assisted segmentation in 3D (ITAS3D).^15^ Here, an image-sequence translation model was trained to convert 3D datasets of prostate tissue, stained with a fluorescent small-molecule analog of hematoxylin and eosin (H&E) to look like 3D immunofluorescence datasets of cytokeratin 8 (CK8), which labels the luminal epithelial cells that define all prostate glands. Subsequently, a CV-based 3D segmentation routine was used to segment out the epithelium and lumen compartments within the gland, as well as the surrounding stromal regions. This multistage ITAS3D pipeline offered several initial advantages, including the ability to leverage a cheap and rapid small-molecule stain while obviating the need for tedious and subjective manual annotations to train a 3D gland segmentation model. However, a shortcoming of ITAS3D is that it was a relatively complex two-step procedure involving deep learning image-sequence translation followed by CV-based segmentation of the resulting “synthetic immunofluorescence” datasets. Furthermore, the CV step often still required manual parameter tweaking that was time consuming and tedious. To overcome these limitations, we sought to train a deep learning model for direct 3D segmentation based on our H&E-analog raw datasets, in which we used our previously generated 3D segmentation masks (generated by ITAS3D) as labels for training.

**Fig. 1:**
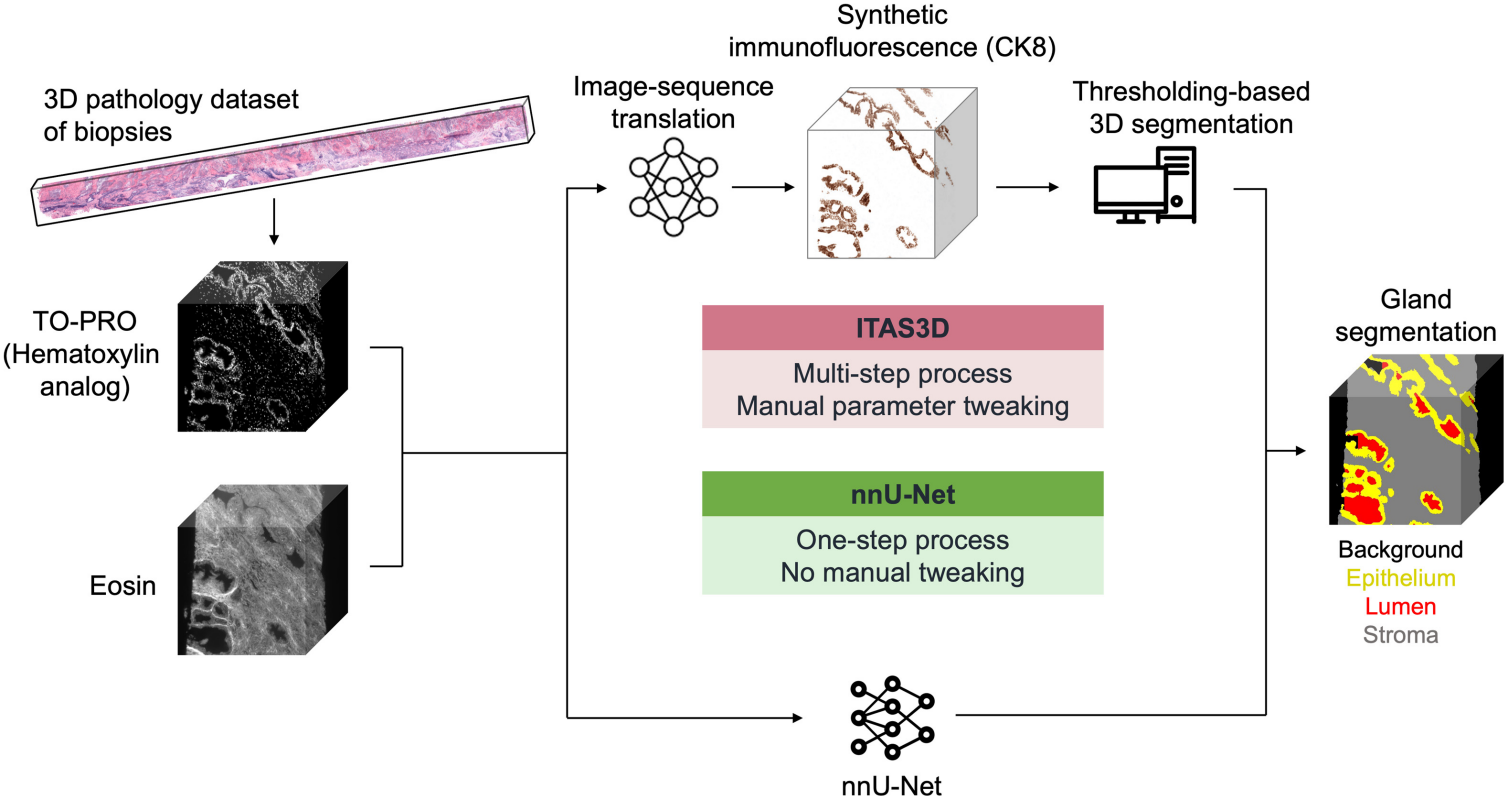
General pipeline comparison between ITAS3D (the upper route) and nnU-Net (the lower route) for 3D prostate gland segmentation.

To train a model for direct 3D segmentation of prostate glands based on our raw H&E-analog datasets, we explored the use of nnU-Net,^29^ a 3D segmentation method designed to handle diverse biomedical imaging datasets. nnU-Net automates the key decisions for designing a successful segmentation pipeline for any given dataset and is available as an out-of-the-box segmentation model for those with limited deep learning experience. The pipeline comparison between ITAS3D and nnU-Net is shown in Fig. 1. Here we quantified the accuracy of nnU-Net as well as its execution speed in comparison with our previous ITAS3D pipeline. We also explored the flexibility of nnU-Net to operate on downsampled datasets that are volumetrically 8X smaller (2X smaller in each dimension) than the original inputs to our ITAS3D method.

## 2 Methods

### 2.1 nnU-Net Model

As mentioned in the Introduction, we trained an nnU-Net model to generate 3D segmentation masks directly from H&E-analog input images (3D pathology datasets). nnU-Net’s network backbone is based on the classical U-Net,^30^ but the great value of nnU-Net is that it provides a comprehensive and automated mechanism to perform preprocessing and postprocessing such that little to no user intervention is required from training to inference. nnU-Net extracts key information directly from the input datasets and determines how to optimize various hyperparameters and other model parameters through internal heuristic rules. The loss functions used in the network are Dice loss and cross-entropy loss. The optimizer is fixed to the Stochastic Gradient Descent with Nesterov momentum. A poly learning rate schedule is utilized to minimize the potential for gradient explosion and to ensure an optimal learning curve. The model architecture that nnU-Net optimized for our datasets is summarized in Fig. S1 in the Supplementary Material, with information regarding convolutional layers, filter sizes, input/output size for each layer, etc.

### 2.2 Training Details

Training datasets were collected with a custom-developed second-generation open-top light-sheet (OTLS) microscope,^31^ which had a lateral resolution of 0.9  μm and a raw pixel spacing of 0.45  μm (Nyquist sampling). For prior studies with ITAS3D, 2X-downsampled datasets were used (0.9-μm pixel spacing) as inputs for the initial image-translation stage of ITAS3D. Then, for the CV-based gland-segmentation stage, the image-translated datasets were downsampled by another 2X (1.8-μm pixel spacing). These levels of downsampling were deemed acceptable for the segmentation of large tissue structures such as prostate glands while minimizing computational times and resources (i.e., each factor of 2X in downsampling reduced the 3D dataset sizes by 8X). The final segmentation masks generated by ITAS3D were also 4X downsampled (1.8-μm pixel spacing) compared with the original images obtained by the OTLS microscope.

**Fig. 2:**
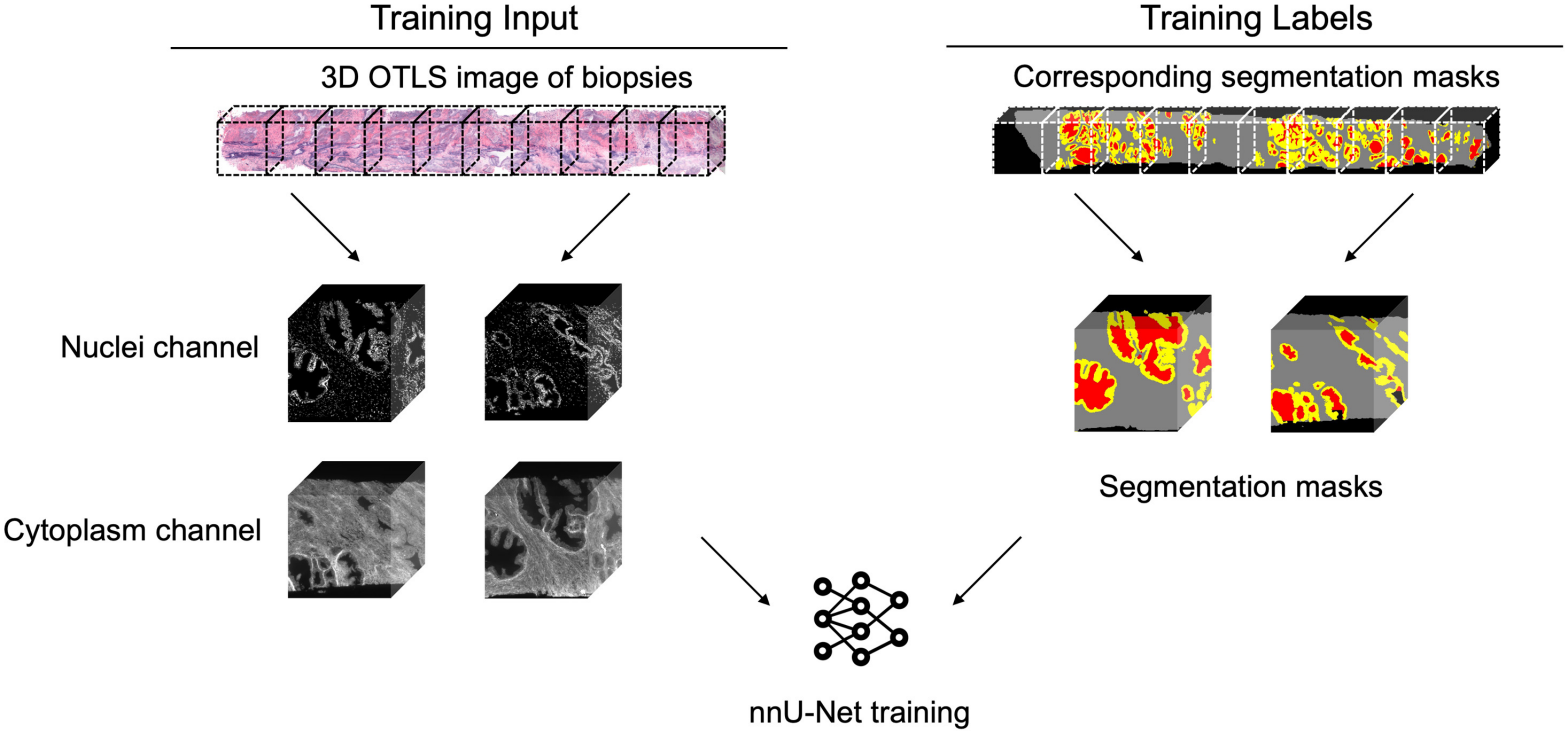
Inputs for training an nnU-Net model from 3D H&E images and paired segmentation masks generated by ITAS3D.

For model training, we first sub-divided the 3D biopsy datasets (∼1  mm×0.7  mm×20  mm) into ∼1×0.7×1  mm blocks (∼512×350×512  pixels at 1.8-μm pixel spacing) to fit within the RAM of our GPUs (Fig. 2). Then, all sub-blocks as well as corresponding segmentation labels were arranged into a folder structure that was appropriate for nnU-Net training.^32^ Note that, during training, nnU-Net internally divides the training dataset in an 80/20 split for training and internal validation, respectively. The actual training session was conducted on a Linux workstation with one NVIDIA RTX 4090 GPU, an AMD Threadripper PRO 5965WX CPU, and 256 GB of RAM.

### 2.3 Inference and External Validation

After training the model, a set of two validation biopsies (sourced from different patients and held out from the training process) were also divided into blocks using the same method as the training biopsies. The trained nnU-Net model was used to infer segmentation masks based on the H&E-analog channels (To-PRO-3 and eosin fluorescence)^15^ of the biopsy datasets, and the inference results were compared against the ITAS3D generated segmentation masks. In addition to the qualitative inspection of the gland segmentation results using nnU-Net, the segmentation masks were quantitatively assessed based on manually annotated 3D segmentation masks (not generated by ITAS3D). A total of 10 tissue volumes from different patients (∼512×512×100  pixels each, representing $∼0.2\text{-}{\mathrm{mm}}^{3}$ of tissue) were generated, and 3D manual annotations (slice by slice) were obtained of the glands (the interface between the epithelium and surrounding stroma) under the guidance of board-certified genitourinary pathologists. These ground-truth manual segmentations enabled us to compare the performance of our model with the original ITAS3D segmentation results and two other baseline segmentation methods, a 2D U-Net^30^ and a 3D watershed^33^ algorithm. The 2D U-Net model was trained on patches derived from 15 regions of interest obtained from five distinct biopsies. The 3D watershed approach begins with a 3D extension of the watershed method, which was applied on the eosin channel to identify candidate lumen regions only. Here, the 3D-watershed algorithm was initiated at marker points that were identified with an Otsu thresholding routine applied on the same eosin-channel images. Likewise, epithelium regions were detected by applying another watershed-based segmentation method on the hematoxylin channels. Candidate lumen regions in which the majority of the boundary pixels were not adjacent to segmented epithelium were eliminated due to the fact that true lumen regions are always enclosed by epithelial cells.

Quantitative evaluation and benchmarking were done by calculating Dice coefficients^34^ and 3D Hausdorff distances^35^ based on the ground truth manual-segmentation masks using a Python package named “seg-metrics.” The Dice coefficient, also known as the Sørensen-Dice coefficient or F1 score, is a similarity metric used to evaluate the agreement between two sets. It is commonly used in the context of image segmentation. The Dice coefficient is defined by the following equation:

$$\mathrm{Dice}(A,B)=\frac{2\times |A\cap B|}{\left|A|+|B\right|},$$ (1)

where A is the first set, B is the second set, |A∩B| is the size of the intersection of sets A and B, |A| is the size of set A, and |B| is the size of set B. The 3D Hausdorff distance is a mathematical measure used to quantify the dissimilarity between two sets of 3D points or shapes. In the context of 3D data, such as point clouds or volumetric representations, the Hausdorff distance measures how far one set of points is from the other, taking into account both the maximum distance of a point in one set to the nearest point in the other set, and vice versa. It provides a way to assess the similarity or dissimilarity between two 3D shapes or structures. Mathematically, the 3D Hausdorff distance is defined as

$$H(A,B)=\max({\text{sup}}_{a\in A}\inf_{b\in B}d(a,b),\sup_{b\in B}\inf_{a\in A}d(b,a)),$$ (2)

where A and B are the two sets of points or shapes in 3D space; a and b represent individual points in sets A and B, respectively; d(a,b) is the distance metric between points a and b; sup denotes the supremum (least upper bound); and inf denotes the infimum (greatest lower bound).

Subsequently, pairwise comparisons were done based on the quantitative measurement results [Figs. 4(c) and 4(d), sample size n=10]. Two-sample t tests were performed to calculate p values for nnU-Net against each benchmarked method (ITAS3D, 2D U-Net, and 3D watershed, respectively) without correction for multiple comparisons.

### 2.4 Speed Comparisons

For speed benchmarking, we recorded the ITAS3D pipeline execution time as well as nnU-Net inference time for three randomly selected biopsies. The average size of the three biopsies was ∼1×0.7×20  mm, which corresponded to ∼1000×700×20000  pixels for 0.9-μm pixel spacing and ∼500×350×10000  pixels for 1.8-μm pixel spacing (after 2X downsampling). For nnU-Net, 3D segmentation masks were generated in a single step. By contrast, the ITAS3D pipeline involved four steps from H&E inputs to 3D segmentation masks: data preprocessing, image translation to CK8, image mosaicking, and CV-based segmentation. For ITAS3D, we excluded any time required for manual parameter tweaking for the CV-based segmentation step. We only recorded the terminal and code execution time for the four steps. All tests were conducted on the same computer to ensure consistent hardware and software/environment configurations.

## 3 Results

### 3.1 Model Training

To train an nnU-Net model, we randomly selected 16 prostate biopsies from the 118 biopsies previously processed by ITAS3D.^15^ The original H&E images taken from our second-generation OTLS microscope^31^ were used as input data (0.9-μm pixel spacing), and the segmentation masks generated by ITAS3D (1.8-μm pixel spacing, verified by board-certified pathologists) were used as corresponding training labels for the gland epithelium, lumen, and stromal compartments. Compared with the input datasets used in our prior ITAS3D gland-segmentation pipeline, the datasets used here were downsampled by 2X in all three dimensions to match the segmentation masks generated by ITAS3D (1.8-μm pixel spacing), resulting in an 8X reduction in dataset sizes and computational resources. For the default 1000 epochs, training took approximately 3 days with the workstation described in the Methods section. The detailed training curves are shown in Fig. S2 in the Supplementary Material.

### 3.2 Qualitative Visual Evaluation

**Fig. 3:**
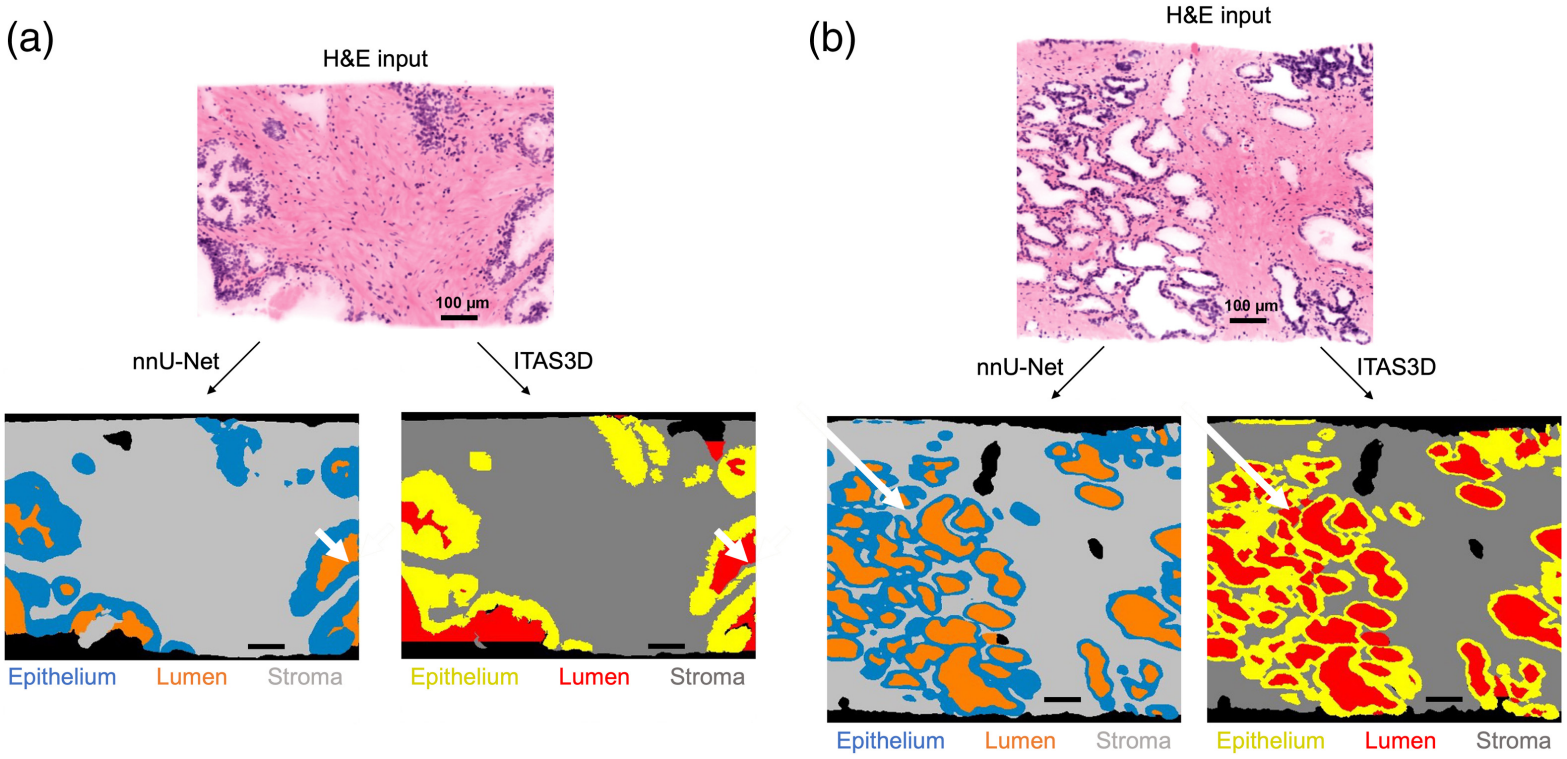
Qualitative evaluation of the trained model’s performance. (a), (b) 2D frames showing side-by-side comparisons between nnU-Net-generated segmentation masks and ITAS3D-generated segmentation masks, (Videos 1 and 2 show 3D datasets of the masks) both from the same H&E image input. The examples shown in panels (a) and (b) are from different tissue samples. Bold white arrows point to regions where nnU-Net outperforms ITAS3D. Scale bars=100  μm (Video 1, MP4, 10.9 MB [URL: https://doi.org/10.1117/1.JBO.29.3.036001.s1]; Video 2, MP4, 10.9 MB [URL: https://doi.org/10.1117/1.JBO.29.3.036001.s2]).

Two prostate biopsies, sourced from distinct patients and not utilized in the training process, were chosen at random for qualitative assessment (visual inspection). These biopsies are a subset of the 118 biopsies previously processed by ITAS3D to generate 3D gland segmentation masks. These masks were used to compare the performance of the nnU-Net model versus the prior ITAS3D pipeline. Although all inputs and outputs are 3D datasets (Videos 1 and 2), we selected example 2D frames for demonstration purposes. Two sets of comparisons are shown in Fig. 3. For each panel, the input H&E image is shown on the top (note that the false-colored^36^ H&E images shown are for demonstration purposes; all computations are performed on our original grayscale 2-channel fluorescence datasets). The nnU-Net inference result is shown on the bottom-left and the ITAS3D segmentation result is shown on the bottom-right. The nnU-Net model exhibits smoother edges compared with the traditional CV-generated masks [white arrow in Fig. 3(a)], but it occasionally misinterprets certain lumen regions as stroma. Figure 3(b) shows how the nnU-Net inference results can occasionally outperform the ITAS3D segmentation masks. For example, as shown by the white arrow in Fig. 3(b), ITAS3D incorrectly labels a stromal region as a lumen region, whereas nnU-Net is more accurate.

### 3.3 Quantitative Evaluation

**Fig. 4:**
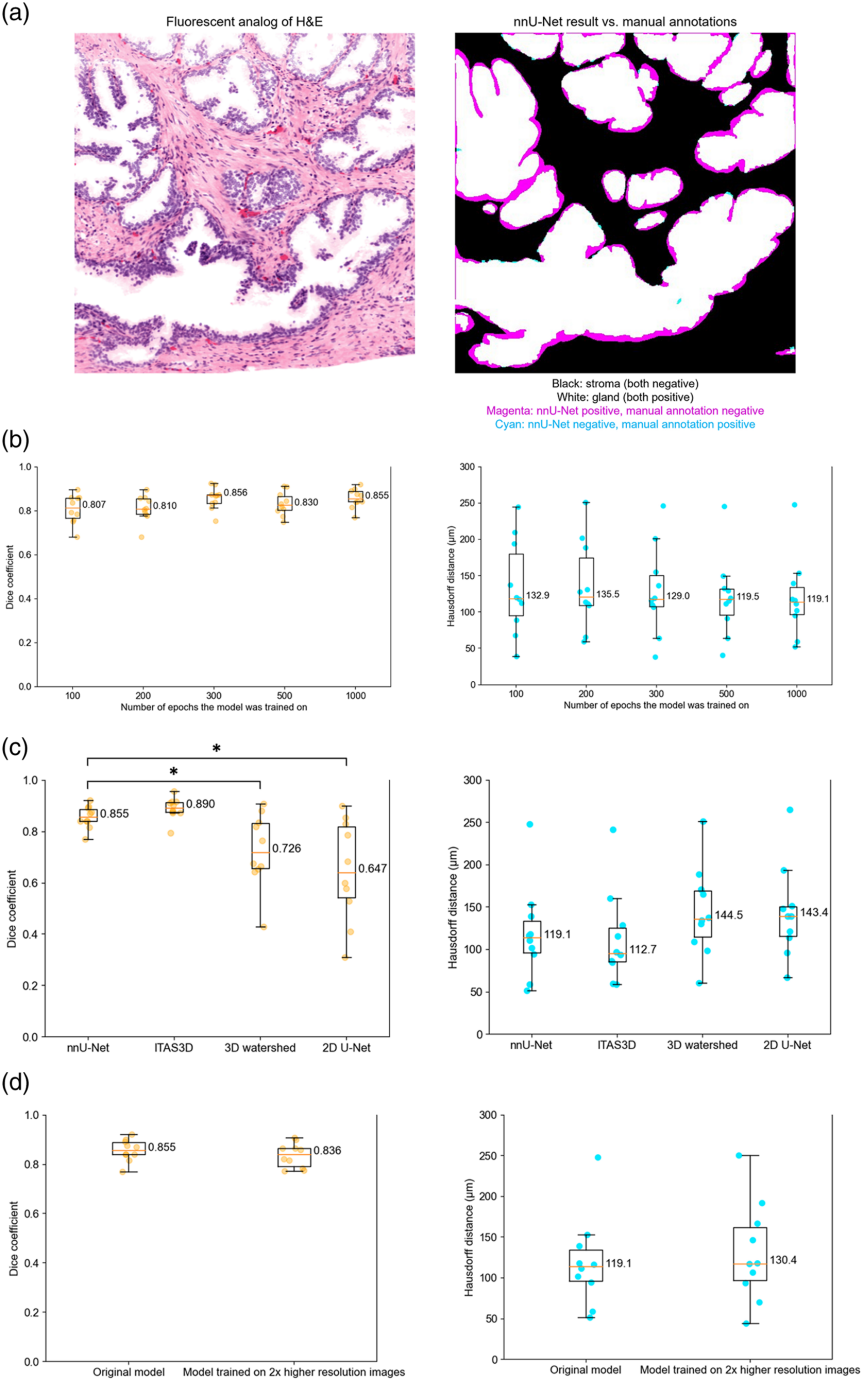
Quantitative measurements of the nnU-Net model’s performance in terms of Dice coefficient and 3D Hausdorff distance as calculated from 10 manually annotated test regions (3D volumetric depth stacks each containing hundreds of manually annotated 2D images) that were not used for training. Asterisk (*) denotes a p value<0.05. (a) Example of a nnU-Net generated segmentation mask versus the manually annotated segmentation mask (Video 3, MP4, 11 MB [URL: https://doi.org/10.1117/1.JBO.29.3.036001.s3]). (b) Benchmark of the model when trained for 100, 200, 300, 500, and 1000 epochs, respectively. (c) Benchmark of nnU-Net method against ITAS3D and other baseline segmentation methods. (d) Benchmark of the original nnU-Net model against a new nnU-Net model trained on datasets with 2X-higher resolution (8X larger size for a 3D dataset).

In addition to the above qualitative examination of the model’s performance, we also conducted a quantitative measurement based on manually annotated gland masks (epithelium plus lumen) of the 3D prostate images. Unlike the segmentation masks generated by ITAS3D, which were employed as training labels for nnU-Net, the manual annotations are a more-authentic ground truth. However, they only delineate the boundaries between the gland epithelium and surrounding stroma (two compartments) rather than delineating all three segmented tissue compartments (epithelium, lumen, and stroma). See Fig. 4(a) for an example visualization of a nnU-Net generated result versus a manually annotated gland-segmentation mask.

#### 3.3.1 Ablation study of training process

During the training process, we did an ablation study to determine the optimal number of epochs for maximizing model performance while adhering to a reasonable training timeframe. In addition to using the nnU-Net’s default trainer, which trains a model for 1000 epochs, we customized four other trainers to run for 100, 200, 300, and 500 epochs, respectively. All trainers employed the same linear-descent technique for learning-rate reduction, transitioning linearly (as a function of epochs) from a rate of 0.01 to 0. Subsequently, all five trained models underwent quantitative benchmarking using the above-mentioned manually annotated validation set. The results shown in Fig. 4(b) demonstrate that the model already provides a good performance when trained for only a few hundred epochs, but the overall performance (both for the Dice coefficient and 3D Hausdorff distance metrics) of the model improves slightly as it is trained for more epochs. We used the 1000-epoch model to perform any related validation tasks.

#### 3.3.2 Benchmarking with ITAS3D and other methods

We used our manual ground-truth annotations to quantitatively compare the nnU-Net model with ITAS3D as well as two other baseline 3D segmentation strategies [Fig. 4(c)]. The Dice coefficient for the nnU-Net masks was calculated across all 10 test cases and ranged from 0.7 to 0.95, with an average of 0.855. The 3D Hausdorff distance across all test cases ranged from 50 to 250  μm, with an average of 119.1  μm. The nnU-Net model is slightly inferior to ITAS3D, as expected, because the nnU-Net model was trained on the segmentation masks provided by ITAS3D. However, their performance is quite comparable, with both of these methods clearly outperforming baseline segmentation methods such as 3D watershed and 2D U-Net.

Pairwise comparisons show that there is a significant performance difference between nnU-Net and both the 2D U-Net and 3D watershed methods, whereas there is no significant difference between nnU-Net and ITAS3D in terms of Dice coefficient. On the other hand, 3D Hausdorff distance measurements demonstrated insignificant pairwise differences between all methods. The 3D Hausdorff distance considers the maximum distance from a point in one set to the closest point in the other set. Therefore, even a single outlier can dramatically increase the Hausdorff distance, masking more subtle differences between segmentation masks. This effect is compounded by the complexity of the shapes and structures in our 3D pathology datasets, which can lead to high variability in Hausdorff distance measurements.

#### 3.3.3 Sensitivity to sampling pitch (image resolution)

As mentioned previously, this study was performed with 3D datasets that were 2X downsampled (8X smaller in size for a 3D image) in comparison with the 3D datasets used as inputs in the original ITAS3D pipeline. To show that this does not cause a significant deterioration in nnU-Net performance, we also trained another model based on the original-resolution (0.9-μm pixel spacing) H&E-analog input datasets that ITAS3D used. Training the model with original-resolution datasets (0.9-μm pixel spacing) took approximately 14 days with the workstation described in the Methods section.

Interestingly, the model trained on the dataset with a larger image size/resolution exhibited a marginal dip in performance compared with the previous model that we worked on [Fig. 4(d)]. In any case, the ability to achieve good segmentation performance with downsampled H&E-analog input datasets (compared with ITAS3D inputs) is beneficial for computational resources and training/inference times.

### 3.4 Speed Benchmarking

**Fig. 5:**
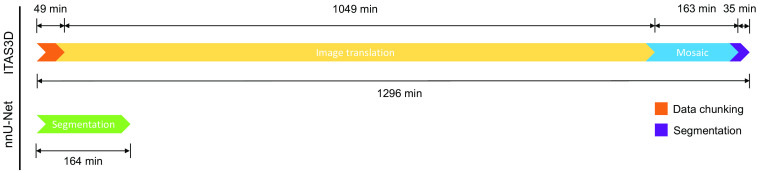
Speed benchmark between nnU-Net and ITAS3D execution with the same PC workstation (see Sec. 2). The ITAS3D timeline excludes the time taken for manual parameter adjustments, which often makes ITAS3D much more time consuming than plotted here. The average physical size of the biopsies used for these benchmarking tests was approximately 1×0.7×20  mm.

The execution time for ITAS3D can be divided into four parts: data chunking, image translation, image mosaicking, and segmentation. In addition, the manual tweaking of parameters is often needed for ITAS3D but is omitted in these calculations as it varies from case to case. Nevertheless, it is worth noting that, in some cases, ITAS3D may require hours of manual effort to achieve satisfying results. The results show that the execution speed of nnU-Net is significantly faster than the original ITAS3D pipeline when performed on identical computing resources (Fig. 5). This is facilitated by the fact that nnU-Net can start with 2X downsampled inputs compared with ITAS3D and is a one-step process. The elimination of manual parameter adjustments further improves the efficiency of the nnU-Net pipeline.

## 4 Discussion

The advent of non-destructive 3D pathology technologies coupled with advances in artificial intelligence have ushered in an era of diagnostic possibilities. AI and machine learning techniques have an important role in the analysis of these large datasets, so pathologists and other investigators can gain insights and gain trust in 3D pathology. Prostate cancer diagnosis and risk assessment have historically relied upon the Gleason grading system, which relies on the interpretation of glandular morphologies seen in 2D histology sections. This approach, however, is hampered by interobserver variability and limitations in correlating Gleason scores with patient outcomes. We are motivated to investigate the ability of 3D pathology datasets to offer a more comprehensive and accurate view of glandular morphologies across much-larger volumes of tissue than are typically assessed via 2D histopathology. Here, we harnessed the power of deep learning to address the task of 3D gland segmentation within prostate biopsies, which is a critical component toward developing machine classifiers of patient risk based on 3D glandular features.

In prior work, we developed an annotation-free pipeline, ITAS3D, for 3D gland segmentation based on tissues stained with a cheap and fast fluorescent analog of H&E staining. With ITAS3D, a deep-learning image-translation method was first used to create synthetic immunolabeled datasets based on H&E-analog input datasets. With synthetic immunolabeling of a CK8 biomarker, which is expressed by the luminal epithelial cells that define all prostate glands, it was then possible to use standard CV methods such as intensity thresholding and hole-filing algorithms to create 3D segmentation masks of the prostate gland lumen regions, epithelial regions, and stromal regions. Having generated hundreds of 3D segmentation masks in an annotation-free manner using ITAS3D, we had the opportunity to train an end-to-end deep-learning model for single-step gland segmentation based on our H&E-analog input datasets.

Our qualitative and quantitative results demonstrate the efficacy of the open-source nnU-Net package, which produces smoother and comparably accurate segmentation results when compared with our prior ITAS3D masks. The Dice coefficient and Hausdorff distance metrics, calculated based on withheld “ground truth” datasets that were manually annotated, were comparable between nnU-Net and ITAS3D. However, it is worth noting that certain imperfections, such as mistaking lumen fields for stromal compartments, were noticed. To address this issue, one potential path is to fine-tune and/or augment the ITAS3D-generated segmentation masks used to train the nnU-Net model. Alternatively, simple and robust CV methods may be useful to post-process and improve the segmentation masks generated by nnU-Net.

One of the most valuable outcomes of our study was the substantial improvement in execution speed offered by nnU-Net compared with the multi-stage ITAS3D pipeline. The efficiency gains achieved by nnU-Net are significant, not only in terms of computational time but also in terms of simplicity and automation compared with the ITAS3D pipeline that can require some manual tuning of segmentation parameters. Because nnU-Net can operate well on 2X-downsampled datasets compared with ITAS3D, this results in a ∼8X reduction in dataset sizes and computational resources. Interestingly, it was found that the model trained on datasets with a larger image size/resolution exhibited a marginal dip in performance (though insignificant through pairwise statistical comparisons). A potential explanation for this is that, because we needed to upsample the ITAS3D segmentation masks as training labels for the higher-resolution nnU-Net model, these up-sampled training labels were not as accurate as true high-resolution segmentation masks. In addition, it is possible that lower-resolution datasets can encourage models to utilize new features that may be more robust.

In conclusion, our study applied nnU-Net as a powerful tool for accurate and efficient 3D gland segmentation within prostate biopsies. With these gland segmentation masks, we and others may be able to extract a diversity of quantitative 3D glandular features (histomorphometric features) to train machine classifiers with the ultimate goal of enhancing prostate cancer risk stratification and treatment decisions. The model’s speed and accuracy will simplify and accelerate future research toward optimizing treatment decisions for individual patients.

## Supplementary Material









## Data Availability

The base code for the nnU-Net method described in this manuscript can be freely accessed through https://github.com/MIC-DKFZ/nnUNet/. The code used for processing the dataset, validation, etc., can be requested from the authors. The original 3D prostate image datasets used in this article (H&E analog datasets and ITAS3D-generated masks) are publicly available on The Cancer Imaging Archive (TCIA) - https://doi.org/10.7937/44MA-GX21.
