# Supplementary material for: Direct three-dimensional segmentation of prostate glands with nnU-Net
Source: J Biomed Opt. 2024 Mar 1;29(3):036001. doi: 10.1117/1.JBO.29.3.036001 (PMC10905031; doi:10.1117/1.JBO.29.3.036001)
Supplement: Supplementary file 1 [file JBO_029_036001_SD001.pdf]

Supplementary information

Title: Direct 3D segmentation of prostate glands with nnU-Net

Authors: Rui Wang,[a] Sarah S.L. Chow,[a] Robert B. Serafin,[a] Weisi Xie,[a] Qinghua Han,[b] Elena Baraznenok[a,b], Lydia Lan[b,c], Kevin W. Bishop[a,b] and Jonathan T.C. Liu[a,b,d,]*
[a]University of Washington, Department of Mechanical Engineering, Seattle, Washington, United States
[b]University of Washington, Department of Bioengineering, Seattle, Washington, United States
[c]University of Washington, Department of Biology, Seattle, Washington, United States
[d]University of Washington, Department of Laboratory Medicine & Pathology, Seattle, Washington, United States

Supplementary figures:

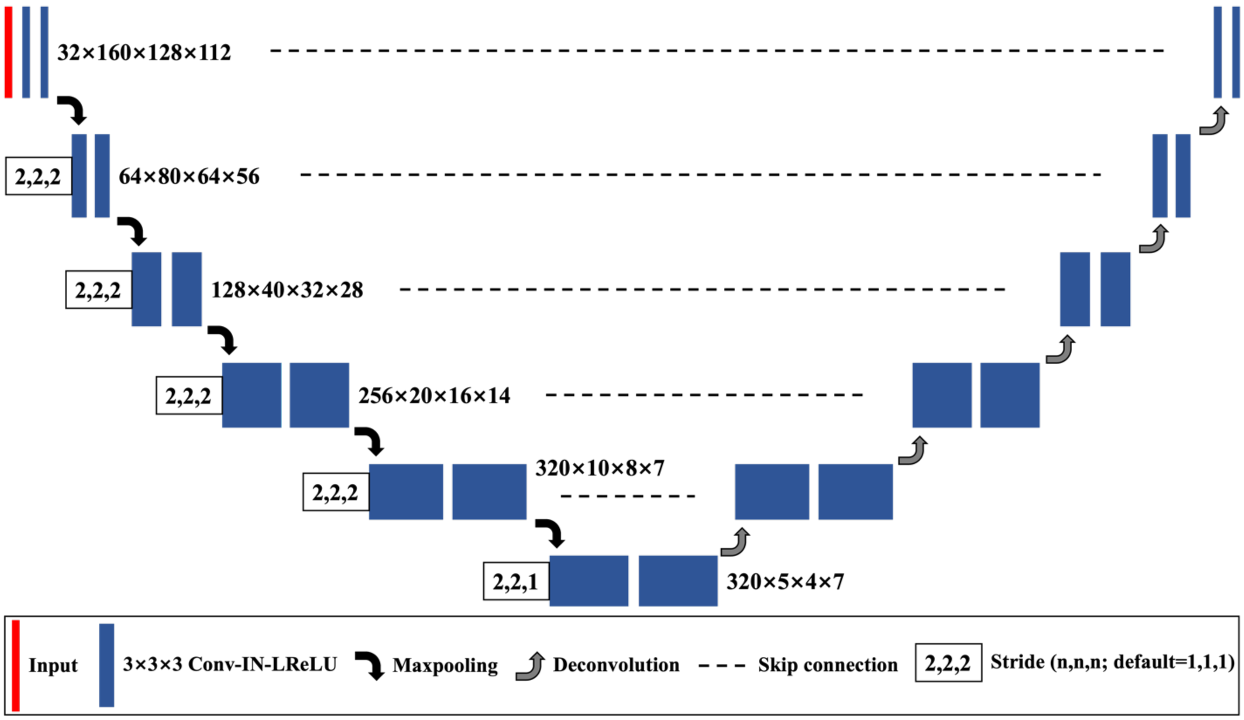

Figure S1. The network architecture generated and optimized by nnU-Net

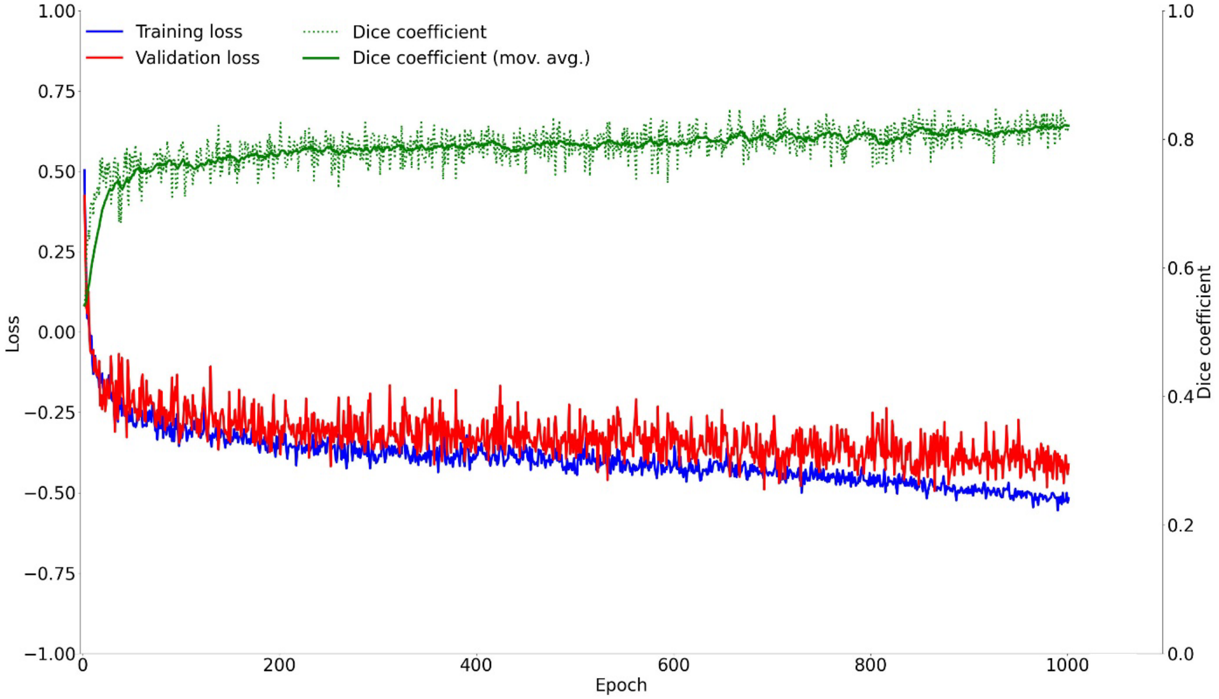

Figure S2. The training curves of the nnU-Net model
